# Translation and Co-translational Membrane Engagement of Plastid-encoded Chlorophyll-binding Proteins Are Not Influenced by Chlorophyll Availability in Maize

**DOI:** 10.3389/fpls.2017.00385

**Published:** 2017-03-28

**Authors:** Reimo Zoschke, Prakitchai Chotewutmontri, Alice Barkan

**Affiliations:** ^1^Max Planck Institute of Molecular Plant PhysiologyPotsdam, Germany; ^2^Institute of Molecular Biology, University of Oregon, EugeneOR, USA

**Keywords:** translation, chloroplast, chlorophyll, ChlH, GUN5, ribosome profiling, maize

## Abstract

Chlorophyll is an indispensable constituent of the photosynthetic machinery in green organisms. Bound by apoproteins of photosystems I and II, chlorophyll performs light-harvesting and charge separation. Due to the phototoxic nature of free chlorophyll and its precursors, chlorophyll synthesis is regulated to comply with the availability of nascent chlorophyll-binding apoproteins. Conversely, the synthesis and co-translational insertion of such proteins into the thylakoid membrane have been suggested to be influenced by chlorophyll availability. In this study, we addressed these hypotheses by using ribosome profiling to examine the synthesis and membrane targeting of chlorophyll-binding apoproteins in chlorophyll-deficient *chlH* maize mutants (Zm*-chlH*). *ChlH* encodes the H subunit of the magnesium chelatase (also known as GUN5), which catalyzes the first committed step in chlorophyll synthesis. Our results show that the number and distribution of ribosomes on plastid mRNAs encoding chlorophyll-binding apoproteins are not substantially altered in Zm*-chlH* mutants, suggesting that chlorophyll has no impact on ribosome dynamics. Additionally, a Zm*-chlH* mutation does not change the amino acid position at which nascent chlorophyll-binding apoproteins engage the thylakoid membrane, nor the efficiency with which membrane-engagement occurs. Together, these results provide evidence that chlorophyll availability does not selectively activate the translation of plastid mRNAs encoding chlorophyll apoproteins. Our results imply that co- or post-translational proteolysis of apoproteins is the primary mechanism that adjusts apoprotein abundance to chlorophyll availability in plants.

## Introduction

Chlorophylls are crucial for the light reactions of photosynthesis. They harvest light energy in the antenna complexes of photosystems I and II (PSI and PSII), they transmit the energy to the reaction centers of both photosystems and they are the primary site of light-induced charge separation. Chlorophylls are tetrapyrroles whose synthesis starts with the reduction of an activated glutamate delivered by the glutamyl-tRNA inside chloroplasts (Masuda and Fujita, 2008; Tanaka et al., 2011). Subsequent steps produce protoporphyrin IX, which is the substrate for the first committed step in chlorophyll synthesis: the insertion of a magnesium ion (Mg^2+^) by the enzyme protoporphyrin IX magnesium chelatase (referred to as magnesium chelatase). Additional reactions generate chlorophyll *a* and its descendant chlorophyll *b*. The majority of chlorophylls are bound by plastid-encoded proteins located in the cores of PSI (PsaA/B) and PSII (PsbA/B/C/D, also known as D1, CP47, CP43, and D2, respectively) and by nuclear-encoded proteins that make up the light harvesting complexes (LHC) (Umena et al., 2011; Croce, 2012; Mazor et al., 2015).

Chlorophylls are highly photoreactive and their accumulation outside the context of a photosynthetic complex produces deleterious reactive oxygen species (Apel and Hirt, 2004). Hence, the synthesis of chlorophylls is coordinated with the availability of chlorophyll-binding apoproteins (Wang and Grimm, 2015). Chlorophyll synthesis is regulated at different steps and activated by light (Brzezowski et al., 2015; Gabruk and Mysliwa-Kurdziel, 2015). In turn, the expression of the nuclear-encoded LHC apoproteins is adjusted by retrograde chloroplast-to-nucleus signaling, which has been suggested to emanate, among other origins, from intermediates in chlorophyll synthesis (Kleine and Leister, 2016; Larkin, 2016). Two of the genes identified in a genetic screen for mutants with disrupted retrograde signaling, *genomes uncoupled* (*gun*) *gun4* and *gun5*, were found to encode components of the chlorophyll synthesis pathway (Susek et al., 1993; Mochizuki et al., 2001). GUN5 constitutes the catalytic H subunit of the magnesium chelatase (ChlH, Mochizuki et al., 2001) and its interaction partner GUN4 enhances the chelatase activity (Adhikari et al., 2011).

The accumulation of plastid-encoded chlorophyll-binding proteins strictly requires chlorophyll (e.g., Klein et al., 1988a; Herrin et al., 1992; Eichacker et al., 1996). Various lines of evidence implicate chlorophyll both as essential for the stability of chlorophyll-binding proteins and as an activator of their synthesis. For example, the results of *in vivo* and *in organello* pulse-labeling assays suggested that the rate of synthesis of chlorophyll-binding apoproteins increases upon a shift from dark to light, coinciding with the onset of chlorophyll synthesis (Fromm et al., 1985; Klein et al., 1988a,b; Malnoë et al., 1988; Mühlbauer and Eichacker, 1998). Furthermore, pulse-labeling experiments with chlorophyll-deficient *Chlamydomonas* and *Synechocystis* cells showed strongly diminished PsbA labeling, suggesting that chlorophyll activates *psbA* translation (Herrin et al., 1992; He and Vermaas, 1998). By contrast, other experiments provided evidence that chlorophyll-binding stabilizes nascent chlorophyll-binding proteins and does not influence their synthesis (Mullet et al., 1990; Herrin et al., 1992; Kim et al., 1994a; Eichacker et al., 1996). Specific ribosome pausing sites were identified on the *psbA* mRNA and were suggested to enable chlorophyll-binding (Kim et al., 1991). However, ribosome pausing was not detectably altered between dark-grown plants and plants illuminated for short periods, arguing against a chlorophyll-mediated pausing mechanism (Kim et al., 1994b). Taken together, the available data provide strong evidence that chlorophyll-binding apoproteins are highly unstable in the absence of chlorophyll, and that several of the apoproteins are synthesized at normal rates in the absence of chlorophyll in barley or *Chlamydomonas* (Mullet et al., 1990; Herrin et al., 1992). Although reduced levels of radiolabeled PsbA in pulse-labeling assays in the absence of chlorophyll suggest that chlorophyll may, in fact, activate translation (Klein et al., 1988a; Herrin et al., 1992; He and Vermaas, 1998), the technical challenge of discriminating lack of protein synthesis from rapid protein turnover in pulse-labeling assays precludes firm conclusions.

The binding of chlorophyll to nascent chlorophyll-binding proteins has also been suggested to be coordinated with their insertion into the thylakoid membrane (Sobotka, 2014). Recently, we have shown that membrane engagement of nascent plastid-encoded chlorophyll apoproteins occurs shortly after the first transmembrane segment emerges from the ribosome (Zoschke and Barkan, 2015). Interestingly, an interaction between a chlorophyll synthesis enzyme and the ALB3 protein translocase in the thylakoid membrane has been demonstrated in cyanobacteria (Chidgey et al., 2014) and this provides a potential mechanism for linking chlorophyll attachment with membrane integration.

Altogether, there is a paucity of firm data that address the interconnection of chlorophyll availability with the synthesis and targeting of plastid-encoded chlorophyll apoproteins. To clarify this issue, we used ribosome profiling to comprehensively analyze (i) ribosome distributions on plastid mRNAs, and (ii) the co-translational membrane-engagement of plastid-encoded proteins in chlorophyll-deficient *chlH* mutants in maize. Our results show that chlorophyll deficiency has little if any effect on the abundance or positions of ribosomes on chloroplast mRNAs, nor on the co-translational membrane engagement of plastid-encoded chlorophyll apoproteins. Together, this implies that plastid apoprotein synthesis and membrane engagement are not regulated by chlorophyll-binding and that changes in protein stability account for adjustments of apoprotein accumulation to chlorophyll levels in plants.

## Materials and Methods

### Plant Material

The Zm*-chlH* mutants were recovered from our large collection of mutants with defects in chloroplast development, the Photosynthetic Mutant Library (Belcher et al., 2015). An Illumina sequencing approach (Williams-Carrier et al., 2010) detected the insertions in the *GUN5/ChlH* ortholog GRMZM2G323024 (B73 genome v.3) in individual yellow seedlings. Gene-specific PCR confirmed that the insertions co-segregated with the phenotype [primers used for genotyping the mutants: et175GRM3230245 5′-gacgaggacacggacaaccta-3′, et1082GRM3230243 5′-ggcgaagttgctggagttg-3′ (Zm*-chlH-1* and Zm*-chlH-2*); et966GRM3230245 5′-CAATTGCTCGGGTGTTTTCA-3′, et1847GRM3230243 5′-AACGAATTGGGGTTGGTGTC-3′ (Zm*-chlH-3*)]. The alleles are recessive and confer a seedling lethal phenotype. Plants were grown in soil in cycles of 16 h light (∼300 μmol m^-2^ s^-1^)/28°C and 8 h dark/26°C. On the eighth day after sowing, leaf tissue was harvested and snap-frozen in liquid nitrogen one hour after the start of the light cycle. Plant tissue was stored at -80°C until use. The second and third leaves to emerge were used for ribosome profiling and chlorophyll measurements whereas the apical half of the second leaf was used for protein extraction and immunoblotting.

### Protein Analysis and Chlorophyll Measurements

SDS–PAGE and immunoblotting used the methods and antibodies described previously (Barkan, 1998; Roy and Barkan, 1998). Chlorophyll content was examined in 80 % acetone by the method described by Porra et al. (1989) and normalized to fresh weight.

### Ribosome Profiling

Microarray-based ribosome and transcriptome profiling experiments were carried out as in Zoschke et al. (2013). Spatially resolved analysis of stromal and thylakoid membrane-tethered ribosomes was performed as in Zoschke and Barkan (2015). For the latter approach, a micrococcal nuclease pre-treatment was performed to remove mRNA-tethered ribosomes from thylakoid membranes before pelleting the membranes (Zoschke and Barkan, 2015). The microarray figures for Zm*-chlH-1/-2* are based on one biological replicate including three technical replicates (**Figures 2, 5, 6**). The wild-type data in **Figures 5, 6** come from two biological replicates including three technical replicates each, and were taken from Zoschke and Barkan (2015) according to the journal guidelines. The values used to generate the plots are available in **Supplementary Datasets S1, S3**. Due to the known difficulties of a reliable quantification of highly abundant RNAs (problem of saturation effects), signals for tRNAs and rRNAs were excluded from the plotting of total RNA (**Figures 2C,F**). To verify the microarray-based ribosome profiling results, ribosome profiling by deep-sequencing was performed with one biological replicate as described by Chotewutmontri and Barkan (2016) with minimal adjustments: ribosomes were pelleted through sucrose cushions by layering 0.82 ml lysate on a 0.33 ml sucrose cushion (1 M sucrose, 0.1 M KCl, 40 mM Tris acetate, pH 8.0, 15 mM MgCl_2_, 10 mM 2-Mercaptoethanol, 100 μg/ml chloramphenicol, and 100 μg/ml cycloheximide) in a 11 mm × 34 mm tube and centrifugation in a Beckman TLA-100.2 rotor for 1.5 h at 55,000 rpm. Reads were aligned to the maize chloroplast genome using Genbank accession X86563.2 and the quality of the footprints was evaluated (**Supplementary Figure S1**). The data are normalized to ORF length (kilobase) per million reads mapping to nuclear genome coding sequences (rpkm). The data used for the plots are provided in **Supplementary Dataset S2**. RNA was extracted from an aliquot of the same tissue homogenate used for ribosome profiling, and used for transcriptome analysis by either microarray or RNA-sequencing as described previously (Zoschke and Barkan, 2015; Chotewutmontri and Barkan, 2016). Each of the abovementioned ribosome profiling experiments used plant tissue from independent mutant plants.

## Results

### Identification of Transposon-induced *chlH* Mutant Alleles in Maize

**FIGURE 1:**
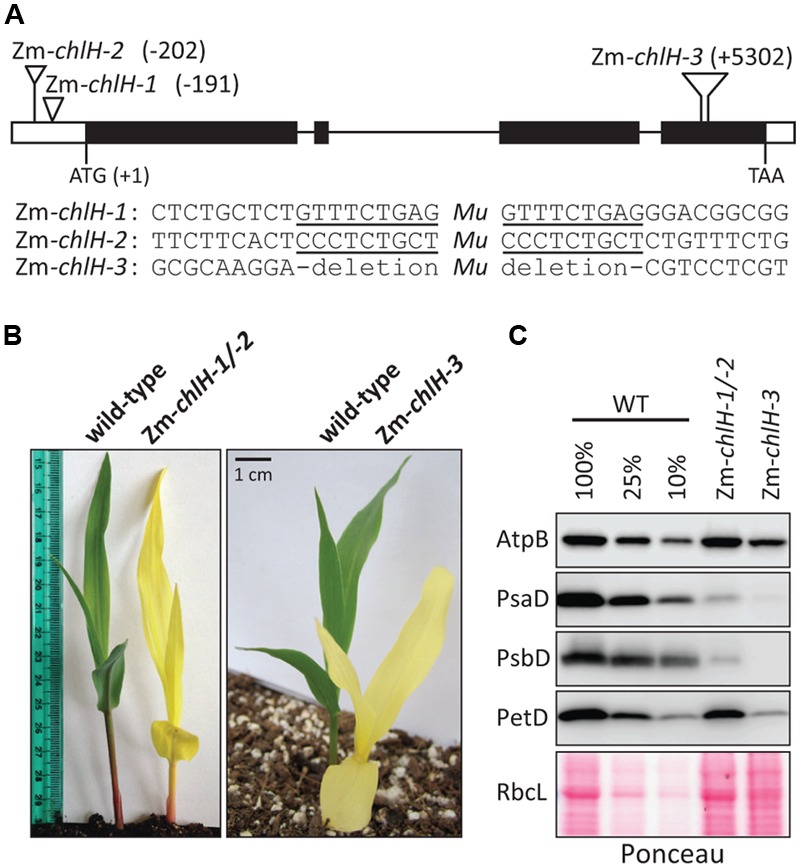
**Overview of Zm*-chlH* mutants. (A)** Sites of *Mu* insertions. The nucleotide position of each insertion with respect to the start codon is indicated. The Zm*-chlH-3* allele has an 11 bp deletion flanking the insertion. The sequences flanking each insertion are shown below the map, with the 9 bp target site duplications underlined. **(B)** Yellow phenotype of Zm*-chlH* mutants. Plants were grown for approximately 8 days in soil. **(C)** Immunoblot analysis of core subunits of photosynthetic complexes. AtpB is a subunit of the ATP synthase, PsaD is a subunit of PSI, PsbD is a subunit of PSII, and PetD is a subunit of the cytochrome *b_6_f* complex. A single blot was probed sequentially with each antibody. The Ponceau S stained blot below illustrates the abundance of RbcL (the large subunit of Rubisco) and also serves as a loading control.

The maize gene encoding the ortholog of *ChlH/GUN5* is designated GRMZM2G323024 in the B73 v.3 genome annotation^1^. We identified three *Mu* transposon insertions in this gene during the systematic sequencing of *Mu* insertions in our large collection of non-photosynthetic maize mutants, the Photosynthetic Mutant Library (Belcher et al., 2015) (**Figure 1A**). Zm*-chlH-1* and Zm*-chlH-2* have insertions in the 5′-UTR, and represent hypomorphic alleles as shown by the reduction of chlorophylls in Zm*-chlH-1/-2* mutants to less than 10% of wild-type levels (**Table 1**). The insertion in Zm*-chlH-3* maps in the last exon and is flanked by a deletion of 11 base pairs. Zm*-chlH-3* is a null allele, based on the facts that chlorophylls are undetectable (**Table 1**) and that the insertion/deletion prevent translation of a highly conserved protein-coding sequence (**Supplementary Figure S2**). All three alleles condition a yellow seedling phenotype (**Figure 1B**). Experiments below used the Zm*-chlH-3* null allele and the heteroallelic progeny of a complementation test cross between Zm*-chlH-1* and Zm*-chlH-2*. Mutants with any of these allele combinations die between the three and four-leaf stage (∼2 weeks after germination), as is typical of non-photosynthetic mutants in maize.

**Table 1:** Chlorophyll content in Zm*-chlH* mutants.

	Chlorophyll per 1 g fresh weight [μg]		
	Chlorophyll *a*	Chlorophyll *b*	Chlorophyll *a* + *b*
wild-type	771.8 ± 80.3	163.4 ± 2.8	935.2 ± 82.9
Zm*-chlH-1/-2*	82.1 ± 12.3	1.7 ± 1.8	83.8 ± 14.1
% wild-type	10.6	1.0	9.0
wild-type	937.6 ± 180.0	193.4 ± 39.4	1131.0 ± 219.4
Zm*-chlH-3*	0.1 ± 0.1	0.1 ± 0.1	0.2 ± 0.2
% wild-type	0.0	0.1	0.0

*Chlorophyll *a* and *b* levels were measured for Zm*-chlH-1/-2* and Zm*-chlH-3* mutants and wild-type siblings as described in Section “Materials and Methods”.*

*Mean values and standard deviations are shown for three biological replicates.*

We examined the abundance of the thylakoid membrane complexes PSII, cytochrome *b_6_f*, PSI, and ATP synthase in the Zm*-chlH* mutants by immunoblot analysis of one core subunit of each complex (**Figure 1C**). The PsaD and PsbD subunits of PSI and PSII, respectively, were reduced more than ten-fold in the hypomorphic mutant and were undetectable in the Zm*-chlH-3* mutant. This is expected based on prior evidence that chlorophyll-binding proteins and the proteins with which they closely associate fail to accumulate in the absence of chlorophyll (e.g., Klein et al., 1988a; Herrin et al., 1992; Eichacker et al., 1996). Interestingly, the PetD subunit of the cytochrome *b_6_f* complex was substantially reduced in the Zm*-chlH-3* null mutant (∼10% of wild-type levels). Reduced levels of the cytochrome *b_6_f* complex were also observed in an *Arabidopsis chlM* mutant (Pontier et al., 2007), and may result from instability of the complex when its single chlorophyll is unavailable (Croce, 2012). The AtpB subunit of the ATP synthase and the large subunit of Rubisco (RbcL) accumulated to normal levels in the hypomorphic mutant but were reduced approximately four-fold in the Zm*-chlH-3* null mutant; the reduction of these proteins is less severe than that of subunits of PSI, PSII, and the cytochrome *b_6_f* complex, consistent with the fact that the ATP synthase and Rubisco lack chlorophyll. It is interesting, however, that AtpB and RbcL were reduced at all, and possible explanations are discussed below.

### Ribosome Placement on Plastid mRNAs Encoding Chlorophyll-binding Apoproteins Is Not Substantially Altered in Zm-*chlH* Mutants

**FIGURE 2:**
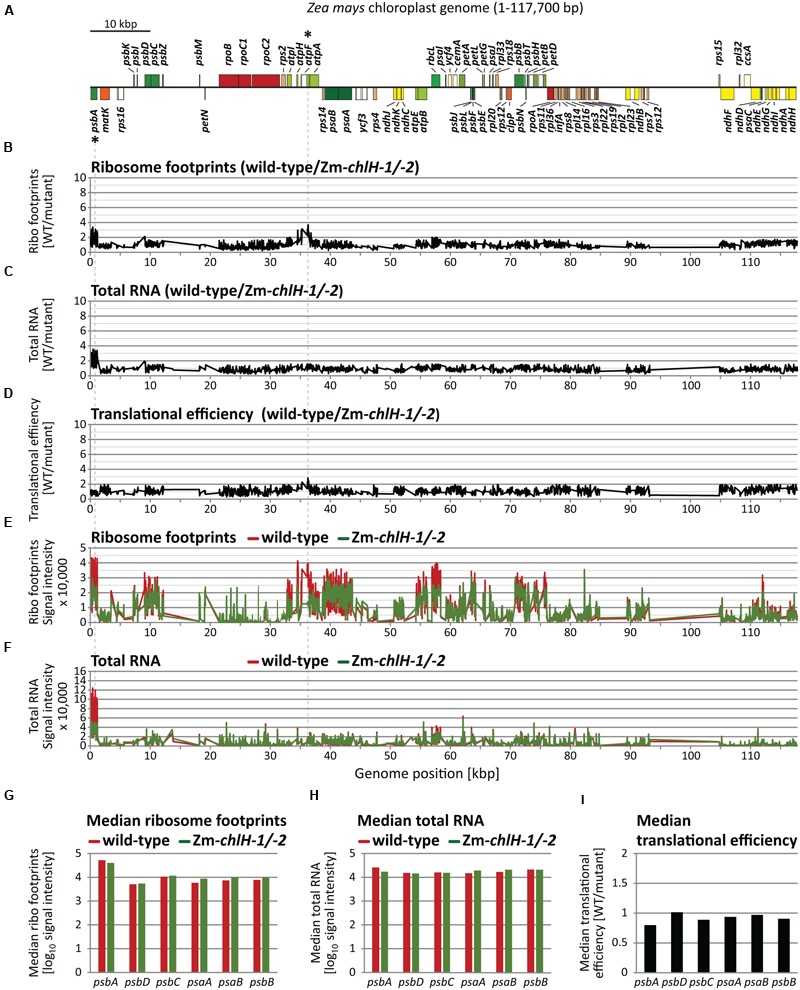
**Microarray-based plastome-wide analysis of ribosome footprint and transcript abundances in the wild-type and the Zm*-chlH-1/-2* mutant.** Plots are based on data that are provided in **Supplementary Dataset S1**. Genome positions refer to the reference maize chloroplast genome (Maier et al., 1995). **(A)** Gene map indicating protein-coding genes of the maize chloroplast genome created with OGDraw (Lohse et al., 2013). The circular map of the chloroplast genome was linearized and shows only the first of the two large inverted repeat regions. Asterisks mark genes with defects in gene expression based on the microarray data in B-F (wild-type to mutant signal ratio > 3). Dashed lines connect these genes on the map with peaks in the plots below. **(B)** Normalized ratios of ribosome footprint signals (Ribo footprints) in wild-type versus mutant are plotted as a function of genome position. Peaks designate regions with more ribosome footprints in the wild-type compared to the mutant. **(C)** Normalized ratios of total RNA signals in wild-type versus mutant are plotted as a function of genome position. Peaks represent regions with higher RNA accumulation in the wild-type compared to the mutant. **(D)** Translational efficiencies were calculated as the ratios of ribosome footprint ratios (shown in **B**) to total RNA ratios (shown in **C**). **(E)** Normalized ribosome footprint signal intensities obtained from wild-type (red) and mutant (green). **(F)** Normalized total RNA signal intensities obtained from wild-type (red) and mutant (green). **(G)** Median ribosome footprint signals for chlorophyll apoprotein-coding ORFs (signals plotted in log_10_-scale). **(H)** Median total RNA signals for chlorophyll apoprotein-coding ORFs (signals plotted in log_10_-scale). **(I)** Median translational efficiency values for chlorophyll apoprotein-coding ORFs.

To address whether chlorophyll alters ribosome behavior on apoprotein-coding mRNAs, we used ribosome profiling to compare the distribution of ribosomes among and within plastid ORFs in wild-type and Zm*-chlH* mutant leaf tissue. The original ribosome profiling method uses deep-sequencing to map and quantify ribosome footprints – small mRNA segments that are protected by ribosomes from nuclease attack (Ingolia et al., 2009). Our initial experiments used a modified method that substitutes high-resolution microarrays for deep-sequencing to profile ribosome footprints (Zoschke et al., 2013; **Figure 2**). We hybridized microarrays to ribosome footprints (**Figures 2B,E**) and total RNA (**Figures 2C,F**) from wild-type and Zm*-chlH-1/-2* samples; translational efficiencies were then calculated as the ratios of ribosome footprints to RNA abundances (**Figure 2D**). Genotype-dependent differences in the abundance of ribosome footprints from several genes are apparent, the largest of which mapped to the *psbA* and *atpF* coding regions (**Figures 2B,E,G**; ratios > 3). However, these result from a difference in mRNA abundance (**Figures 2C,F,H**). A several-fold decrease in *psbA* mRNA had previously been observed in other maize mutants with diverse chloroplast biogenesis defects and is, therefore, likely to result from pleiotropic effects of the photosynthesis defect (Zoschke et al., 2013; Williams-Carrier et al., 2014). The calculated translational efficiencies for all ORFs encoding chlorophyll-binding apoproteins (**Figure 2I**), and in fact for all other ORFs, were very similar in the wild-type and the Zm*-chlH-1/-2* mutant (**Figure 2D**). These results strongly suggest that there are no substantive differences between the wild-type and the Zm*-chlH* mutant in the number of ribosomes bound per mRNA for the plastid-encoded chlorophyll apoproteins or any other chloroplast ORF.

**FIGURE 3:**
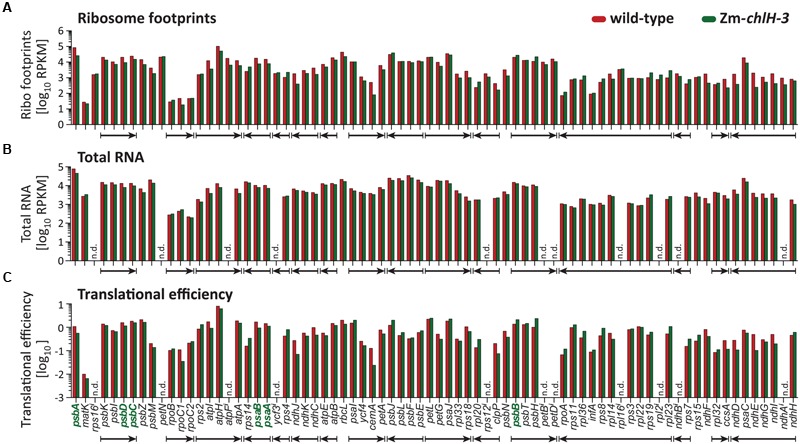
**Summary of deep-sequencing analysis of plastid ribosome footprint and transcript abundances in wild-type and Zm*-chlH-3* mutant leaf tissue.** Genes encoding chlorophyll-binding proteins are shown in bold green font. The data are displayed as the number of reads per ORF after normalizing to ORF length (kilobase) per million reads mapping to nuclear genome coding sequences (RPKM; values are shown in **Supplementary Dataset S2**). Translational efficiencies are calculated as the ratios of ribosome footprint to transcript reads. Co-transcribed genes are marked with arrows according to the direction of transcription. **(A–C)** Ribosome footprint abundance, transcript levels, and the derived translational efficiencies are displayed according to native gene order on the maize chloroplast genome. RNA levels and translational efficiencies of *petN* and intron containing ORFs (marked with i) were not determined (n.d.) due to technical limitations that preclude accurate quantification of the mRNAs. The ribosome footprint values provided for intron-containing ORFs come only from the last exon or, in the case of *rps12*, from exon 2.

To validate and expand on these findings, we repeated the experiment by using deep-sequencing to profile ribosome footprints. Deep-sequencing offers greater sensitivity than the microarray approach and is especially well suited for detecting changes in ribosome distribution within an ORF at codon resolution. We used the null mutant Zm*-chlH-3* for this experiment to ensure that the trace amounts of chlorophyll present in the Zm*-chlH-1/2* mutants used for the microarray experiment did not mask any effects that chlorophyll might have on ribosome behavior. The normalized abundance of ribosome footprints mapping to each chloroplast gene is plotted in **Figure 3**. Translational efficiencies were calculated by normalizing ribosome footprint abundance to RNA abundance (**Figure 3C**). Unlike the Zm*-chlH-1/2* mutant, four genes (*cemA, ndhE, ndhJ*, and *rpoC1*) showed more than three-fold decrease of translational efficiency in the Zm*-chlH-3* mutant compared to wild-type. However, as observed by microarray analysis of the Zm*-chlH-1/2* mutant, no substantial differences in translational efficiency of mRNAs encoding chlorophyll apoproteins were detected between wild-type and the Zm*-chlH-3* mutant.

**FIGURE 4:**
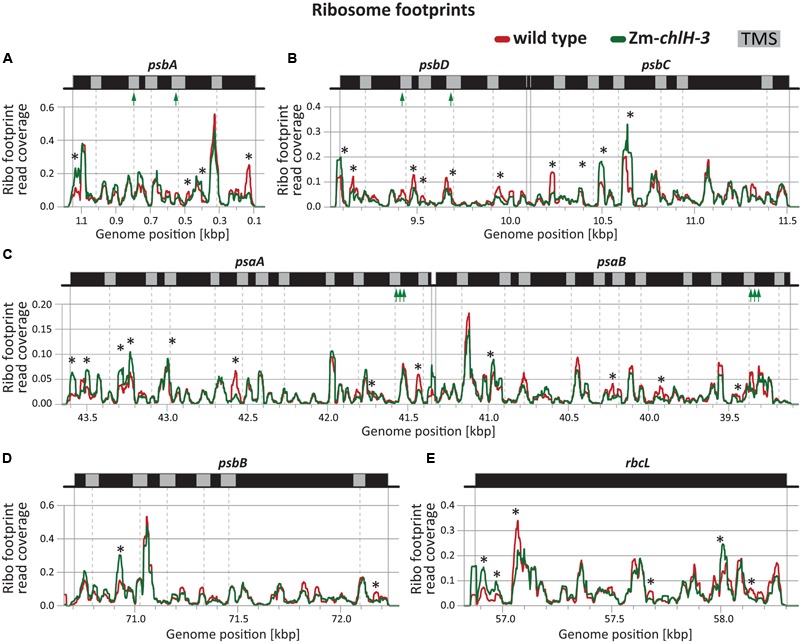
**Ribosome footprint distributions along mRNAs encoding chlorophyll apoproteins based on deep-sequencing data.** Data from wild-type and Zm*-chlH-3* mutant plants are plotted in red and green, respectively. Annotations are as in **Figure 2**. Total read counts within the genomic region shown in each panel of each genotype were standardized to a value of 100 (based on coverage normalized to million reads mapping to nuclear coding sequences). The positions of annotated transmembrane segments (TMS) and chlorophyll-binding sites are shown by gray rectangles and green arrows, respectively. TMS positions are based on information provided previously (Zoschke and Barkan, 2015). Asterisks denote minor differences in ribosome coverage between wild-type and Zm*-chlH-3* (ratio > 2 or < 0.5). **(A**–**D)** Normalized ribosome footprint distributions along mRNAs encoding chlorophyll binding apoproteins. **(E)** Normalized ribosome footprint distribution along the *rbcL* mRNA is shown as a control. *rbcL* encodes the large subunit of Rubisco, which does not bind chlorophyll.

The dynamics of ribosome movement along an ORF are reflected by the relative abundance of ribosomes at each codon, with longer ribosome dwell times resulting in a higher abundance of ribosome footprints (Ingolia, 2014). To determine whether chlorophyll impacts ribosome pausing, we analyzed the distribution of ribosomes along plastid mRNAs encoding chlorophyll-binding apoproteins (**Figure 4**). The profiles of peaks and valleys in these ribosome coverage plots are very similar between the mutant and wild-type, suggesting that the deficiency of chlorophyll does not substantially alter pausing at specific sites or the relative rates of initiation and elongation. Minor differences in ribosome distribution were detected between wild-type and the Zm*-chlH-3* mutant at several positons and may indicate chlorophyll-dependent changes in apoprotein translation behavior (more than two-fold diminished or increased ribosome occupancy is marked by asterisks in **Figures 4A–D**). However, similar features were found for many other reading frames that do not code for chlorophyll-binding proteins (**Figure 4E** shows *rbcL* as an example). Consequently, these differences are not likely to be a direct consequence of the presence or absence of chlorophyll on ribosome dynamics.

In sum, our results show that the distribution of ribosomes among and within ORFs encoding chlorophyll apoproteins is not markedly altered in Zm*-chlH* mutants. This provides strong evidence that chlorophyll does not act as a specific regulator of the synthesis of plastid-encoded chlorophyll apoproteins.

### Co-translational Membrane Engagement of Nascent Chlorophyll-binding Apoproteins Is Not Altered in a Zm-*chlH* Mutant

**FIGURE 5:**
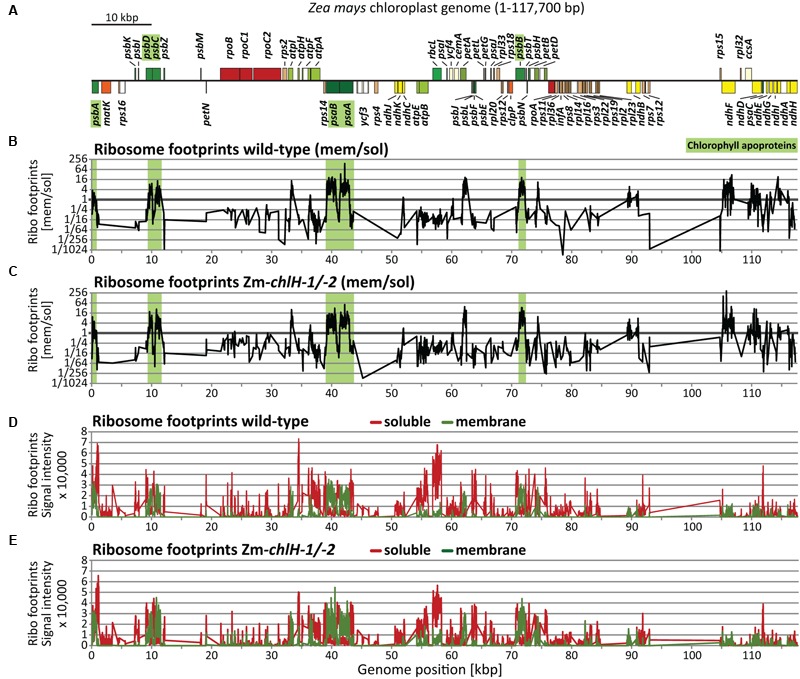
**Plastome-wide analysis of co-translational membrane engagement in wild-type and Zm*-chlH-1/-2* mutant plants by microarray-based ribosome profiling. (A)** Map of the maize chloroplast genome showing only protein-coding genes. Genes highlighted in green encode chlorophyll-binding apoproteins. Plots are based on data that are provided in **Supplementary Dataset S3**. Plots and data for wild-type-derived footprints are identical to those we presented previously (Zoschke and Barkan, 2015). **(B,C)** Normalized ratios of ribosome footprint signals from membrane and soluble fractions in wild-type **(B)** and mutant **(C)** leaf tissue, plotted according to genome position. Note that ribosomes that are tethered to membranes solely by mRNA are recovered in the soluble fraction (see **Supplementary Figure S3**). Green shaded regions mark ORFs encoding chlorophyll-binding apoproteins. **(D,E)** Normalized signals for soluble (red) and membrane-bound (green) ribosome footprints in wild-type **(D)** and mutant **(E)** leaf tissue.

To address whether chlorophyll availability impacts the co-translational engagement of chlorophyll-binding apoproteins with the thylakoid membrane, we used a previously described approach that reports the partitioning of ribosome footprints between the membrane and soluble fractions (**Supplementary Figure S3**); this method reveals the point in nascent peptide synthesis at which co-translational membrane engagement occurs (Zoschke and Barkan, 2015). We isolated ribosome footprints from separated membrane and soluble fractions of the Zm*-chlH-1/2* mutant and examined them by competitive hybridization to our maize chloroplast microarrays (**Figure 5**). The results did not reveal any substantial difference in co-translational membrane engagement of nascent thylakoid proteins in the Zm*-chlH* mutant compared to wild-type plants (**Figures 5B,C**). We observed the same set of proteins to be co-translationally membrane-engaged (including the chlorophyll apoproteins; shown as green shaded regions in **Figures 5B,C**), and the relative signal intensities of ribosome footprints recovered from membrane and soluble fractions are similar in Zm*-chlH* mutant and wild-type plants (**Figures 5B–E**).

**FIGURE 6:**
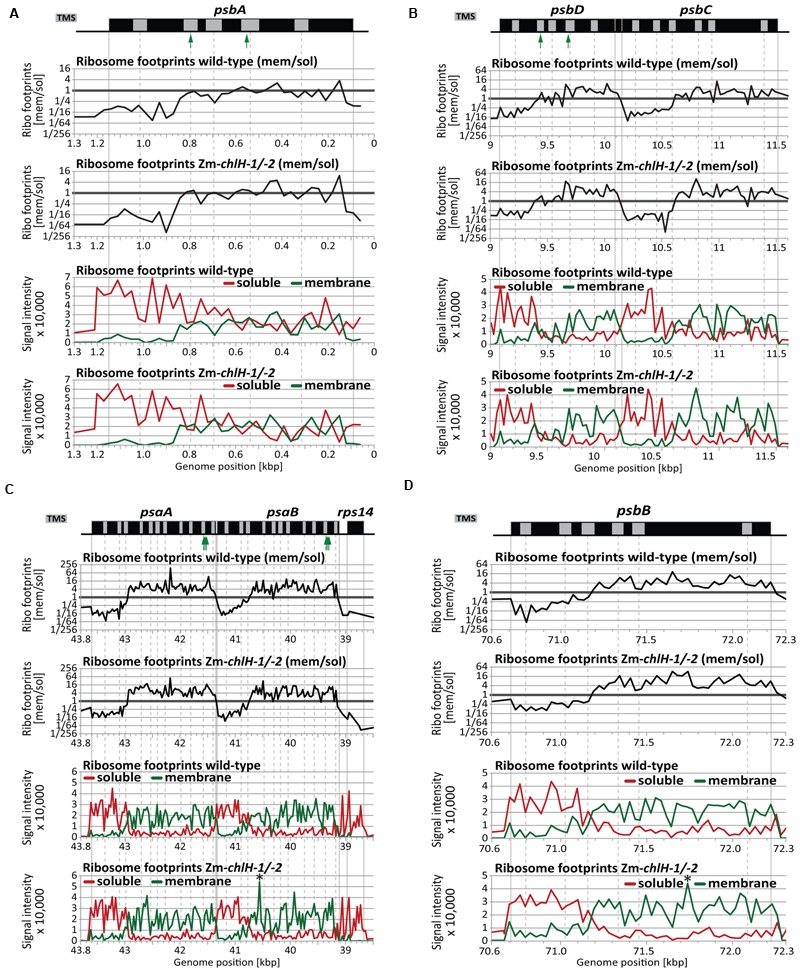
**Zoom-in views of the co-translational membrane engagement of chlorophyll-binding apoproteins in wild-type and Zm*-chlH-1/-2* mutant plants.** Gene maps are drawn to scale above the plots. The positions of annotated transmembrane segments (TMS) and chlorophyll-binding sites are shown by gray rectangles and green arrows, respectively. TMS positions are based on information provided previously (Zoschke and Barkan, 2015). The plots of wild-type data were taken from Zoschke and Barkan (2015). The upper two plots in each panel show normalized ratios of ribosome footprint signals from membrane and soluble fractions of plants of the indicated genotype. The lower two plots in each panel show the signals for membrane (green) and soluble (red) ribosome footprints in plants of the indicated genotype. Asterisks denote minor differences in ribosome coverage between wild-type and Zm*-chlH-1/-2* (see Results). **(A)** Co-translational membrane engagement of the nascent chlorophyll apoprotein PsbA (D1). **(B)** Co-translational membrane engagement of the nascent chlorophyll apoproteins PsbD (D2) and PsbC (CP43) encoded by the overlapping *psbD* and *psbC* reading frames. **(C)** Co-translational membrane engagement of the nascent chlorophyll apoproteins PsaA and PsaB encoded by the adjacent *psaA* and *psaB* genes. Data from the *rps14* gene, which is co-transcribed with *psaA* and *psaB*, is included to illustrate the origin of the soluble ribosome footprints derived from the *psaB* 3′-UTR. **(D)** Co-translational membrane engagement of the nascent chlorophyll apoprotein PsbB (CP47).

High-resolution views of the same data (**Figure 6**) showed that the point at which membrane engagement of nascent chlorophyll apoproteins takes place is very similar between wild-type and Zm*-chlH* mutant plants (**Figures 6A–D**). Furthermore, the topographies of the mutant plots closely resemble those of the wild-type plots, providing further evidence that chlorophyll has little if any effect on the dynamics of ribosome movement through these ORFs. Minor isolated differences were detected for several probes: e.g., peaks were observed for membrane-attached ribosome footprints in the *psaB* and *psbB* coding regions in the Zm*-chlH* mutant that were absent in the wild-type (marked by asterisks in **Figures 6C,D** bottom panels). This might reflect ribosome pauses that differ between wild-type and mutant. However, we favor the view that these differences result from technical variations because we did not detect analogous changes in ribosome distribution when profiling unfractionated chloroplast lysates (**Figures 2B,E, 4C,D**).

Altogether, our results demonstrate that chlorophyll availability does not impact the initial co-translational engagement of plastid-encoded chlorophyll apoproteins with the thylakoid membrane. However, our assay does not address any possible effects of chlorophyll on the integration of downstream transmembrane segments.

## Discussion

### Chlorophyll-independent Translation of Plastid-encoded Chlorophyll Apoproteins

Compared to the sophisticated knowledge about the structure of the photosystems and the location of chlorophylls therein (Umena et al., 2011; Croce, 2012; Mazor et al., 2015), little is known about the coordination of apoprotein synthesis with chlorophyll availability. Although it is well established that chlorophyll binding proteins do not accumulate in the absence of chlorophyll (e.g., Klein et al., 1988a; Herrin et al., 1992; Eichacker et al., 1996), the relative contributions of increased protein instability and reduced protein synthesis remain unclear. In this study, we used three different ribosome profiling approaches (each employing independent mutant tissue) to comprehensively analyze the translation of chloroplast mRNAs in chlorophyll-deficient *chlH* maize mutants. The results consistently showed no substantial effect of chlorophyll deficiency on the abundance or distribution of ribosomes on plastid mRNAs encoding chlorophyll apoproteins. These findings strongly argue against a chlorophyll-dependent regulation of the synthesis of plastid-encoded chlorophyll apoproteins in plants.

This interpretation of our data relies on the assumption that ribosome footprint abundance is a valid proxy for rates of protein synthesis, an assumption that is widely made when interpreting ribosome profiling data (Ingolia, 2014). This view is based on a considerable body of evidence that gene-specific differences in translation rate under any particular condition generally result from differences in the rate of translation initiation (e.g., Shah et al., 2013; Hersch et al., 2014). The global rate of translation elongation can be modulated by environmental inputs (Shalgi et al., 2013), but examples of gene-specific differences in translation elongation rates on mRNAs that are native to the host organism are rare. An example of particular relevance to the question we address here involves a nascent peptide that can modulate ribosome stalling at a specific site in response to the small molecule arginine (Fang et al., 2004). It has been suggested that ribosome pause sites may facilitate the co-translational binding of chlorophyll (Kim et al., 1991), but our results do not address that possibility. However, the fact that ribosome distributions along ORFs encoding chlorophyll-binding apoproteins are unaltered in the Zm*-chlH* mutants provides strong evidence against site-specific effects of chlorophyll on ribosome stalling. Our results are consistent with previous findings from experiments with isolated barley chloroplasts, which showed that the chlorophyll apoproteins PsbA, PsbD, and PsaA indeed can be synthesized independent from chlorophyll (Kim et al., 1994a).

The fact that the abundance of Rubisco and ATP synthase subunits are reduced in the Zm*-chlH-3* mutant suggests a global decrease in translation rate in the mutant chloroplasts. Our data are consistent with the possibility that the mutants experience a global reduction in the rates of translation initiation and elongation in the chloroplast such that the distribution of ribosomes within and among genes shows only minor variations. Validation of this possibility and investigation of the underlying mechanism are potential subjects of future investigation. That said, our data do provide strong evidence against any selective effect of chlorophyll on the translation of open reading frames encoding chlorophyll apoproteins.

Altogether, our data strongly support the idea that, in plants, the adjustment of apoprotein accumulation to chlorophyll levels is mainly achieved by co- or post-translational proteolysis of apoproteins when they are not bound by their chlorophyll cofactors. Indeed, it has been suggested that chlorophyll-deficient apoproteins may incorrectly fold or assemble into complexes and thereby trigger their rapid proteolytic turnover (e.g., Kim et al., 1994a; Eichacker et al., 1996). In line with that, binding of chlorophyll can induce folding and assembly of LHC chlorophyll *a*/*b*-binding proteins *in vitro* (Paulsen et al., 2010). Several thylakoid membrane proteases have been assigned to chlorophyll apoprotein processing and homeostasis and are candidates for a proteolytic adjustment of apoprotein levels to chlorophyll availability (van Wijk, 2015; Nishimura et al., 2016). It is important to note that different synthesis and assembly mechanisms apply for PsbA during biogenesis and repair (Jarvi et al., 2015). Since we studied translation in seedlings containing primarily “biogenic” tissue, we cannot rule out that chlorophyll may regulate *psbA* translation during the D1 repair cycle.

### Chlorophyll Is Not Required for the Co-translational Membrane Engagement of Nascent Chlorophyll-binding Apoproteins

The chlorophyll apoproteins PsaA/B and PsbA/B/C/D engage the thylakoid membrane co-translationally (e.g., Kim et al., 1994a; Zoschke and Barkan, 2015; see also **Figures 5, 6**). Assuming co-translational binding of chlorophyll to nascent apoproteins and a coupling to apoprotein folding and membrane integration, it can be speculated that chlorophyll availability may influence the co-translational integration of nascent apoproteins. However, our analysis of ribosome footprints in separated membrane and soluble fractions showed that the position at which the nascent chlorophyll apoproteins engage the thylakoid membrane is not influenced by chlorophyll deficiency. This is perhaps unsurprising, given that none of the chlorophyll interaction sites is located upstream of the first transmembrane segment (UniProt annotations: PsaA (P04966), PsaB (P04967), PsbA (P48183), PsbD (P48184); and Croce, 2012), which comprises the signal that initially engages the membrane (Zoschke and Barkan, 2015). In line with that, the terminal chlorophyll synthesis enzymes and carrier were found to be associated with the thylakoid membrane, which would enable chlorophyll attachment to apoproteins only after membrane engagement of the nascent apoproteins (Wang and Grimm, 2015). It remains possible that the integration of downstream located transmembrane segments that occurs subsequent to chlorophyll attachment is, in fact, influenced by chlorophyll availability in a way that does not change ribosome progression, a possibility that cannot be addressed by ribosome profiling technologies.

## Author Contributions

RZ and AB designed the research; RZ and PC performed the research; RZ, PC, and AB analyzed the data; RZ and AB wrote the paper.

## Conflict of Interest Statement

The authors declare that the research was conducted in the absence of any commercial or financial relationships that could be construed as a potential conflict of interest.

## Footnotes

1 http://cas-pogs.uoregon.edu/#/pog/17223

## Supplementary Material

FIGURE S1 **Characteristics of ribo-seq data demonstrate that the reads derive from bona fide ribosome footprints. (A)** Distributions of sequencing read sizes within the coding sequences of chloroplasts, mitochondria and cytosol are similar to previously published distributions of ribosome footprint sizes (Chotewutmontri and Barkan, 2016). **(B)** Metagene analysis of cytosolic reads around start and stop codons of all nuclear genes indicates specific occupancy of the reads to the coding region. These reads exhibit 3-nucleotide periodicity depicting the characteristic codon movement of ribosomes. Number of reads were normalized per million reads mapped to nuclear coding sequences. **(C)** Comparison of RNA-seq and ribo-seq read coverages for a representative chloroplast transcription unit (coding for *atpI*/-*H*/-*F*/-*A*). RNA-seq reads map to the entire transcription unit whereas ribo-seq reads map specifically to the coding sequences as expected for ribosome footprints.

FIGURE S2 **Ribosome footprint distribution along the Zm*-chlH* mRNA.** Ribosome footprint reads aligning to the Zm*-chlH* gene were normalized to the reads mapping to nuclear coding sequences and are displayed per million for wild-type and Zm*-chlH-3* mutant plants (note the different y-axis scales of the diagrams). A dashed vertical line indicates the *Mu* transposon insertion site in Zm*-chlH-3* to illustrate the absence of translation downstream of the transposon. The about ten-fold reduced ribosome footprint coverage upstream of the transposon insertion is caused by a reduced accumulation of the Zm*-chlH* transcript in the mutant (as detected in our transcript dataset).

FIGURE S3 **Model for the co-translational membrane engagement of nascent chlorophyll-binding apoproteins and its resulting spatially resolved ribosome profiling data (modified from Zoschke and Barkan, 2015). (A)** Ribosomes translating chlorophyll-binding apoproteins become attached to the membrane in a nuclease-resistant fashion by co-translational thylakoid membrane engagement of the nascent peptide. This occurs shortly after the co-translational exposure of a transmembrane segment from the ribosome (see Zoschke and Barkan, 2015). The supposed co-translational binding of chlorophyll (Chl) is indicated. Ribonuclease pre-treatment releases translating ribosomes to the stroma if they are tethered to the membrane in an mRNA-mediated manner (scissors represent RNase-facilitated ribosome release). A hypothetical membrane channel is shown for illustration only and is not intended to imply any particular membrane insertion mechanism. **(B)** Results of the spatially resolved ribosome profiling analysis of stromal and thylakoid membrane-bound ribosomes for the co-translational membrane engagement shown in **A**. Top panel: the signals of soluble (red line) and membrane-bound (green line) ribosome footprints predominate the 5′- and 3′-regions of the reading frame, respectively. Bottom panel: The ratio of membrane to soluble ribosome footprint signals reverses between the 5′- and 3′-end of the reading frame.

DATASET S1 **Data set of microarray-based ribosome profiling and total RNA control experiments illustrated in **Figure 2**.** Data were processed and analyzed as previously described (Zoschke et al., 2013).

DATASET S2 **Data set of deep-sequencing-based ribosome profiling and total RNA control experiments displayed in **Figures 3, 4**.** Data were processed and analyzed as previously described (Chotewutmontri and Barkan, 2016).

DATASET S3 **Data sets of spatially resolved microarray-based ribosome profiling experiments shown in **Figures 5**, 6.** Data were processed and analyzed as previously described (Zoschke and Barkan, 2015). The wild-type data were taken from Zoschke and Barkan (2015).
