# Supplementary figures and images for: Translation and Co-translational Membrane Engagement of Plastid-encoded Chlorophyll-binding Proteins Are Not Influenced by Chlorophyll Availability in Maize
Source: Front Plant Sci. 2017 Mar 28;8:385. doi: 10.3389/fpls.2017.00385 (PMC5368244; doi:10.3389/fpls.2017.00385)

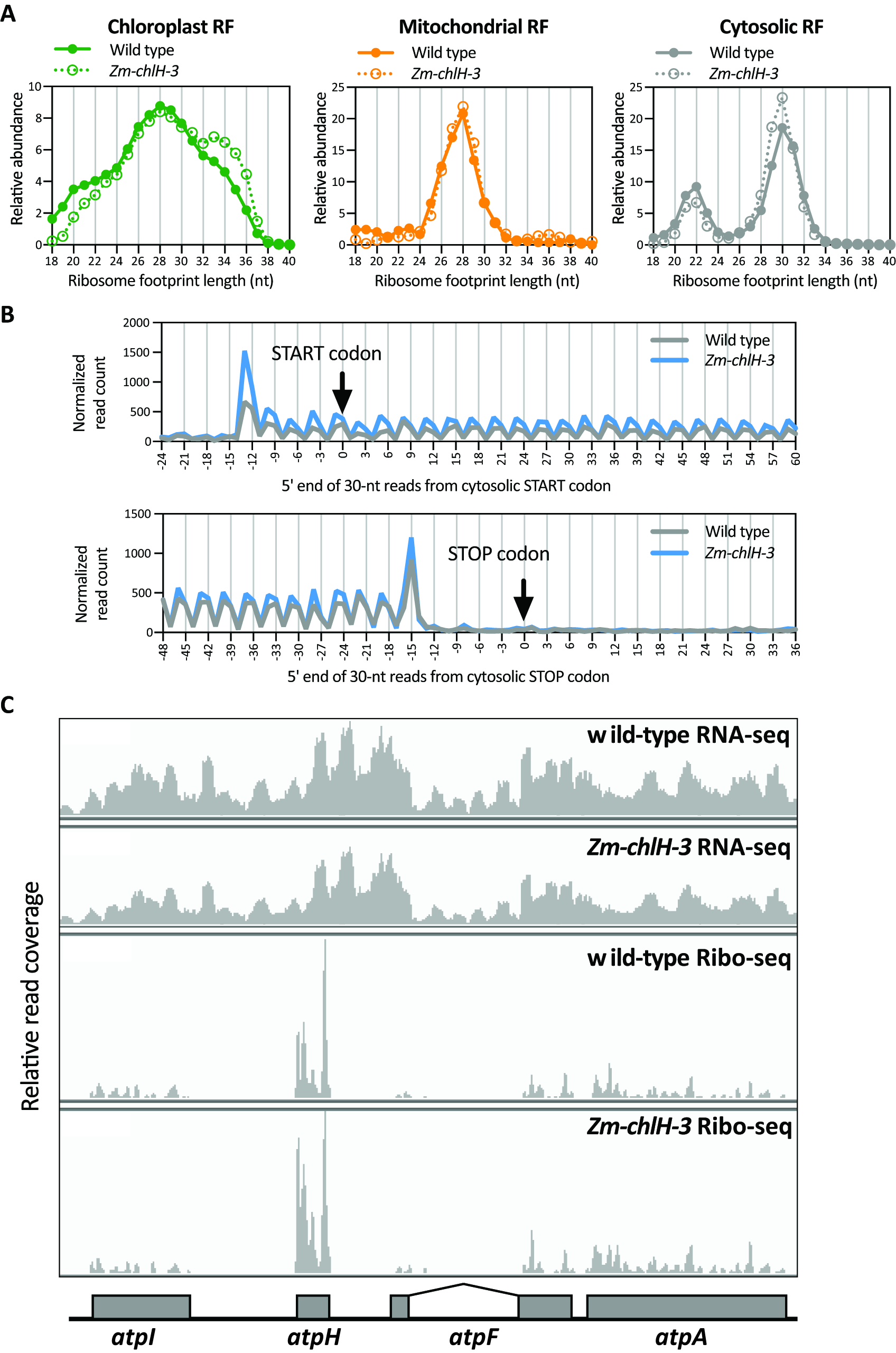

Supplement: FIGURE S1 — Characteristics of ribo-seq data demonstrate that the reads derive from bona fide ribosome footprints. (A) Distributions of sequencing read sizes within the coding sequences of chloroplasts, mitochondria and cytosol are similar to previously published distributions of ribosome footprint sizes (Chotewutmontri and Barkan, 2016). (B) Metagene analysis of cytosolic reads around start and stop codons of all nuclear genes indicates specific occupancy of the reads to the coding region. These reads exhibit 3-nucleotide periodicity depicting the characteristic codon movement of ribosomes. Number of reads were normalized per million reads mapped to nuclear coding sequences. (C) Comparison of RNA-seq and ribo-seq read coverages for a representative chloroplast transcription unit (coding for atpI/-H/-F/-A). RNA-seq reads map to the entire transcription unit whereas ribo-seq reads map specifically to the coding sequences as expected for ribosome footprints. [file Image_1.TIF]

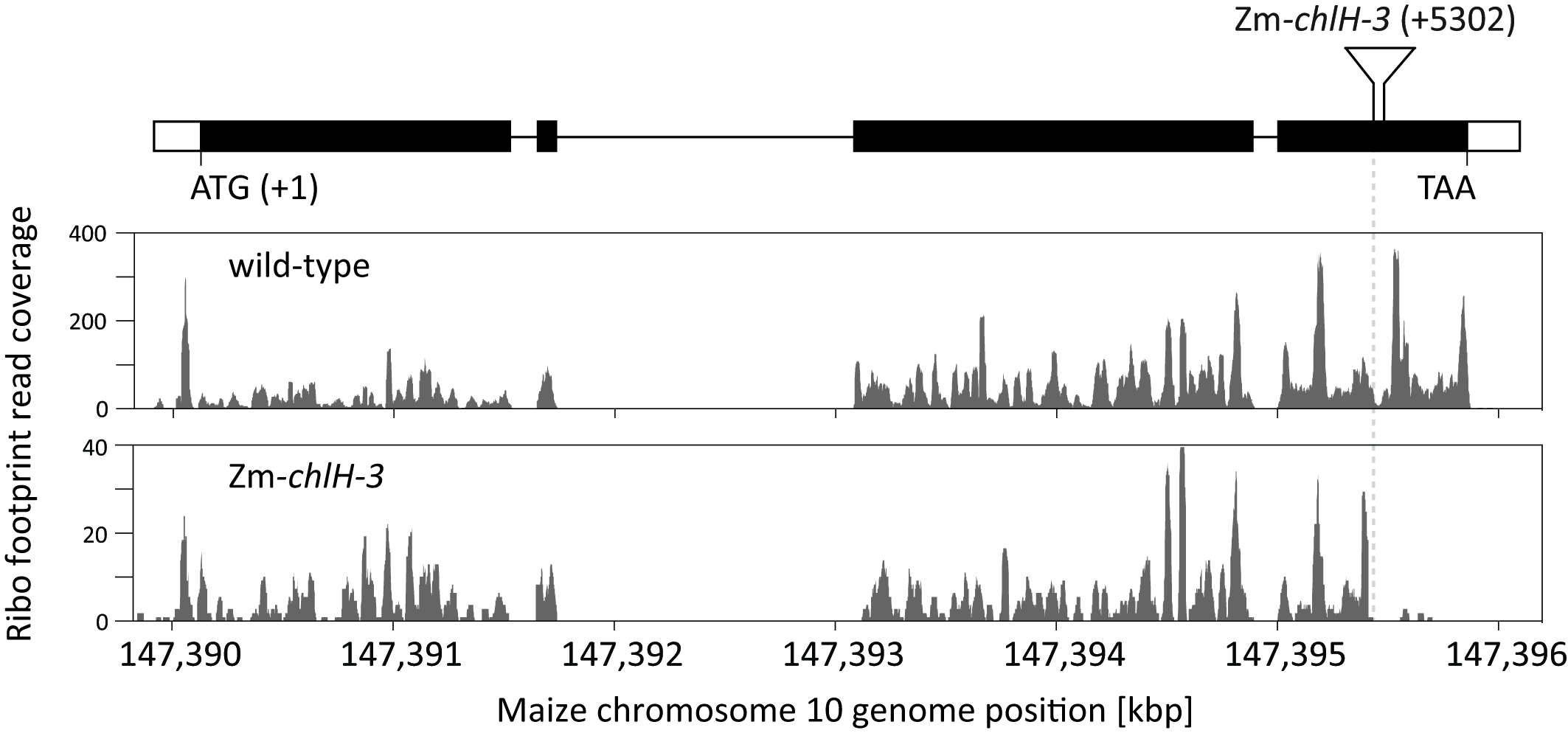

Supplement: FIGURE S2 — Ribosome footprint distribution along the Zm-chlH mRNA. Ribosome footprint reads aligning to the Zm-chlH gene were normalized to the reads mapping to nuclear coding sequences and are displayed per million for wild-type and Zm-chlH-3 mutant plants (note the different y-axis scales of the diagrams). A dashed vertical line indicates the Mu transposon insertion site in Zm-chlH-3 to illustrate the absence of translation downstream of the transposon. The about ten-fold reduced ribosome footprint coverage upstream of the transposon insertion is caused by a reduced accumulation of the Zm-chlH transcript in the mutant (as detected in our transcript dataset). [file Image_2.TIF]

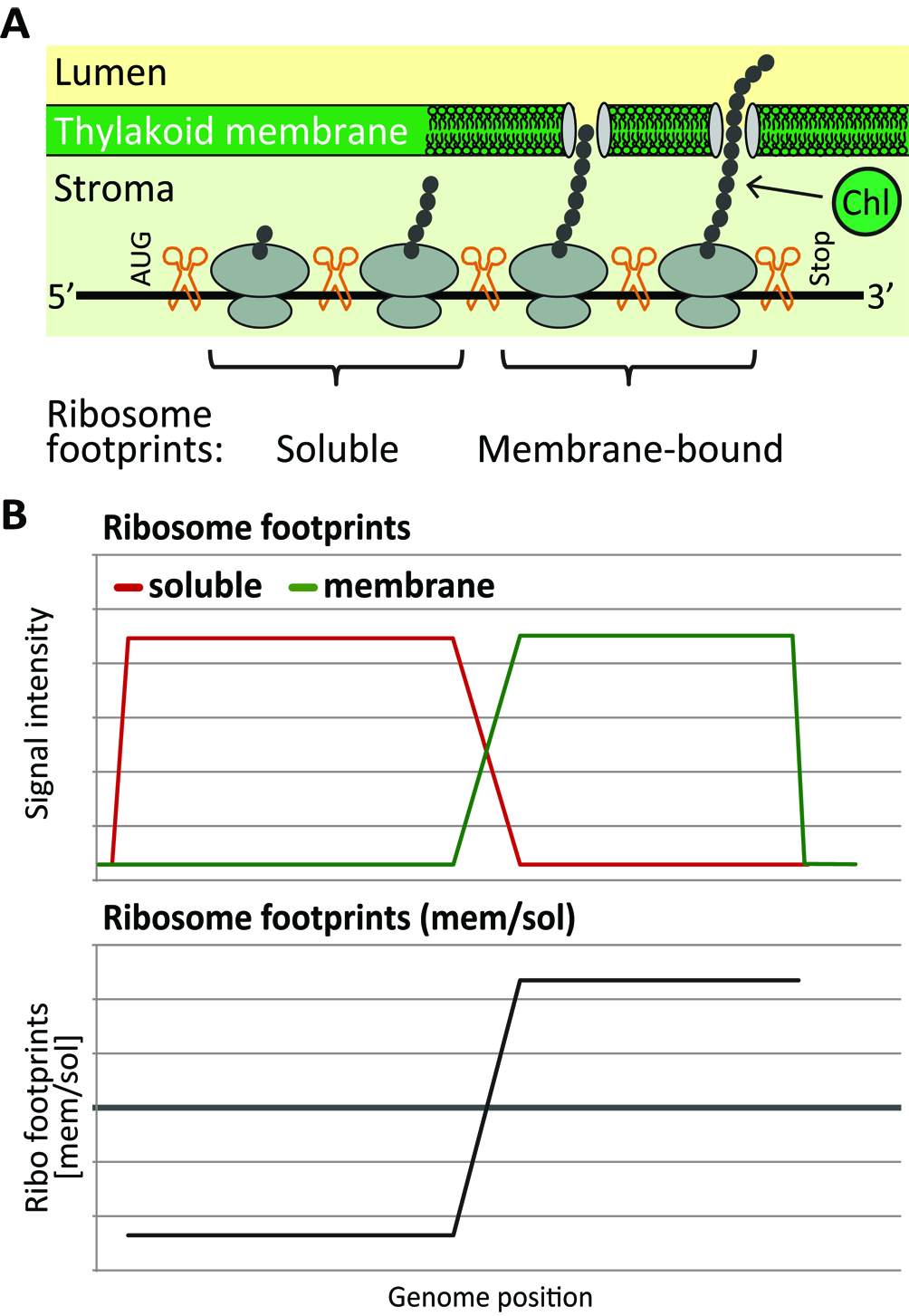

Supplement: FIGURE S3 — Model for the co-translational membrane engagement of nascent chlorophyll-binding apoproteins and its resulting spatially resolved ribosome profiling data (modified from Zoschke and Barkan, 2015). (A) Ribosomes translating chlorophyll-binding apoproteins become attached to the membrane in a nuclease-resistant fashion by co-translational thylakoid membrane engagement of the nascent peptide. This occurs shortly after the co-translational exposure of a transmembrane segment from the ribosome (see Zoschke and Barkan, 2015). The supposed co-translational binding of chlorophyll (Chl) is indicated. Ribonuclease pre-treatment releases translating ribosomes to the stroma if they are tethered to the membrane in an mRNA-mediated manner (scissors represent RNase-facilitated ribosome release). A hypothetical membrane channel is shown for illustration only and is not intended to imply any particular membrane insertion mechanism. (B) Results of the spatially resolved ribosome profiling analysis of stromal and thylakoid membrane-bound ribosomes for the co-translational membrane engagement shown in A. Top panel: the signals of soluble (red line) and membrane-bound (green line) ribosome footprints predominate the 5′- and 3′-regions of the reading frame, respectively. Bottom panel: The ratio of membrane to soluble ribosome footprint signals reverses between the 5′- and 3′-end of the reading frame. [file Image_3.TIF]
